# Coagulation markers and echocardiography predict atrial fibrillation, malignancy or recurrent stroke after cryptogenic stroke: Erratum

**DOI:** 10.1097/MD.0000000000014433

**Published:** 2019-02-01

In the article, “Coagulation markers and echocardiography predict atrial fibrillation, malignancy or recurrent stroke after cryptogenic stroke”,^[1]^ which appears in Volume 97, Issue 51 of *Medicine*, there were edits missed in the article.

The caption for Figure 1 should be “Emory Clinic recommended diagnostic testing for cryptogenic stroke.”

The section starting with “Baseline characteristics of ESUS patients who underwent MOCHA testing…” should be “Baseline characteristics of ESUS patients who underwent MOCHA testing (n = 42) were similar to patients who did not undergo MOCHA testing earlier in our study except that those tested were younger (60 vs 67 years, p = 0.04), less likely to have coronary artery disease (7 vs 27%, p = 0.01) and previous ischemic stroke (10 vs 27%, p = 0.01) and shorter duration of follow-up [median 400 (IQR 151–553) vs 538 (IQR 397–730) days, non-parametric test p = 0.001) (Table 1).”

“ROC analysis showed that abnormal MOCHA markers (AUC = 0.72) and elevated LAVI (AUC = 0.69) had higher discriminative power for the detection of AF than left atrial diameter (AUC = 0.50) (Fig. 3)” should reference Fig. 2 instead.

“We measured levels of each marker comparing patients with AF or malignancy to those with none of the composite outcome (Fig. 2)” should refer to Fig. 3 instead.

**Figure 2:**
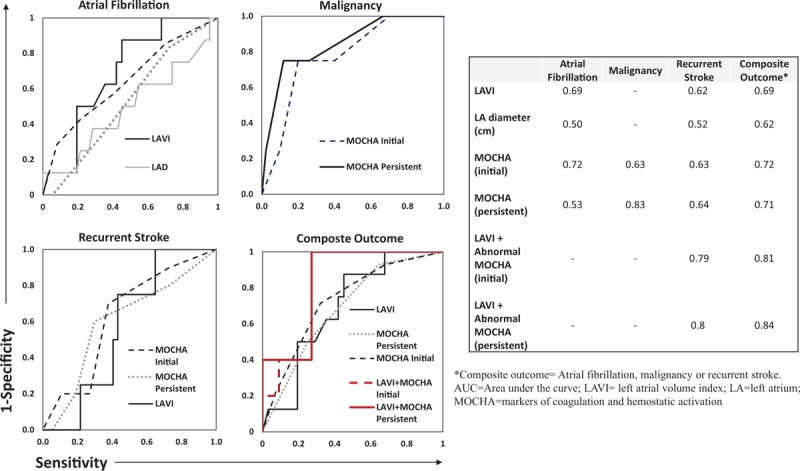
Receiver operator curve analysis of left atrial structural measures, MOCHA levels and endpoints. MOCHA = markers of coagulation and hemostatic activation.

**Figure 3:**
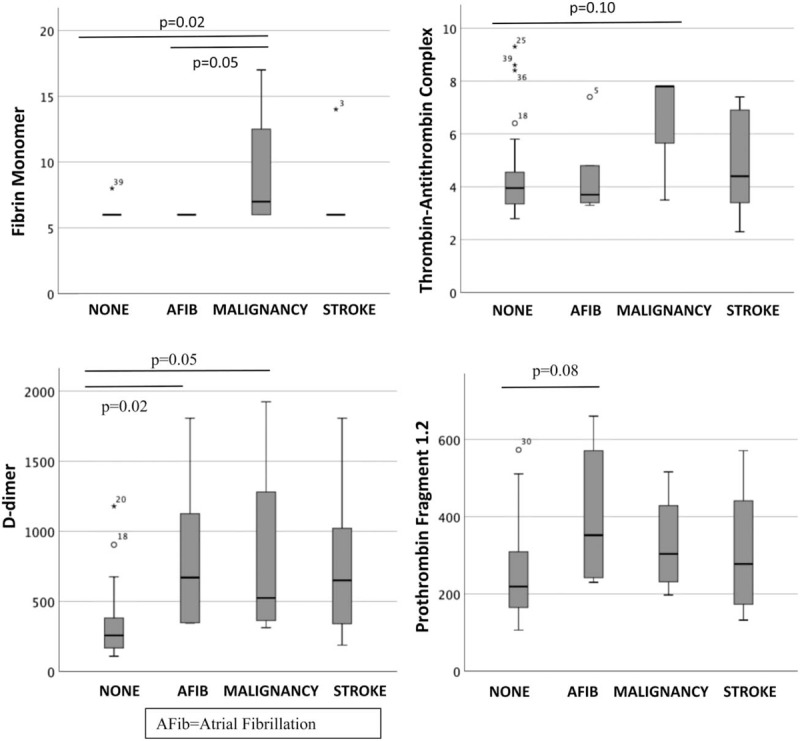
Markers of coagulation and hemostatic activation (MOCHA) levels across the outcome measures.

The p-values should be = instead of < throughout the article.

Below are Figures 2 and 3 with their corrected captions.
